# Root hair phenotypes influence nitrogen acquisition in maize

**DOI:** 10.1093/aob/mcab104

**Published:** 2021-08-06

**Authors:** Patompong Saengwilai, Christopher Strock, Harini Rangarajan, Joseph Chimungu, Jirawat Salungyu, Jonathan P Lynch

**Affiliations:** 1 Department of Plant Science, The Pennsylvania State University, University Park, PA 16802, USA; 2 Department of Biology, Faculty of Science, Mahidol University, Rama VI Road, Bangkok 10400, Thailand; 3 Center of Excellence on Environmental Health and Toxicology (EHT), Ministry of Education, Bangkok, Thailand

**Keywords:** Ammonium, maize, nitrate, nitrogen, roots, root hairs, *SimRoot*, *Zea may*s

## Abstract

**Background and Aims:**

The utility of root hairs for nitrogen (N) acquisition is poorly understood.

**Methods:**

We explored the utility of root hairs for N acquisition in the functional–structural model *SimRoot* and with maize genotypes with variable root hair length (RHL) in greenhouse and field environments.

**Key Results:**

Simulation results indicate that long, dense root hairs can improve N acquisition under varying N availability. In the greenhouse, ammonium availability had no effect on RHL and low nitrate availability increased RHL, while in the field low N reduced RHL. Longer RHL was associated with 216 % increase in biomass and 237 % increase in plant N content under low-N conditions in the greenhouse and a 250 % increase in biomass and 200 % increase in plant N content in the field compared with short-RHL phenotypes. In a low-N field environment, genotypes with long RHL had 267 % greater yield than those with short RHL. We speculate that long root hairs improve N capture by increased root surface area and expanded soil exploration beyond the N depletion zone surrounding the root surface.

**Conclusions:**

We conclude that root hairs play an important role in N acquisition. We suggest that root hairs merit consideration as a breeding target for improved N acquisition in maize and other crops.

## INTRODUCTION

Suboptimal nitrogen (N) availability is a major constraint for crop production, especially in smallholder farming where chemical fertilizers are unaffordable or unavailable (Azeez *et al.*, 2006;Heffer and Prud’homme, 2009). Conversely, large-scale commercial farms utilize a considerable amount of N fertilizer to obtain economically satisfactory yields (Howarth *et al.*, 2002;Ladha *et al.*, 2005;Sutton *et al.*, 2011). It is generally recognized that more than half of applied N is not taken up by crop plants but volatilizes, runs off or leaches away from farmlands causing air and water pollution (Cassman *et al.*, 2002;Janzen *et al.*, 2003;Sheldrick *et al.*, 2002;Baker *et al.*, 2011). Improving crop N acquisition is one of the keys to sustainably increasing and maintaining crop production while preserving environmental quality (Tilman *et al.*, 2002;Lynch, 2007, 2019).

Plant acquisition of soil resources is largely determined by the mode of transport of nutrients from the soil mass to the root surface: diffusion or transpiration-driven mass flow. Whether a nutrient moves through the soil primarily via diffusion or mass flow depends largely upon the concentration gradient and the mobility of the nutrient in the soil (Barber, 1995). For example, immobile nutrients like phosphorus (P), ammonium (NH_4_^+^) and potassium (K) move to the root surface by diffusion, while water-soluble anions like nitrate (NO_3_^−^) and sulphate (SO_4_^2−^) move through the soil matrix by mass flow (Lynch, 2019). Consequently, uptake of sparingly soluble nutrients such as P and K at the root surface may exceed the rate of diffusion in soil, resulting in depletion zones surrounding the roots. Root phenes (phenes are elements of a phenotype) that expand the soil volume subject to the depletion of nutrients through diffusive flux to the root surface can, therefore, increase nutrient acquisition. Root hairs, subcellular extensions of root epidermal cells, are beneficial in this regard.

Long root hairs facilitate P acquisition under low P conditions in both computer simulations (Bouldin, 1961;Ma *et al.*, 2001) and greenhouse and field-grown crops, including maize (Zhu *et al.*, 2010), common bean (Miguel *et al.*, 2015), wheat and barley (Gahoonia and Nielsen, 1997;Gahoonia *et al.*, 1997) and *Arabidopsis* (Bates and Lynch, 2000). Additionally, root hairs release P-mobilizing exudates to enhance P availability in the rhizosphere (Ryan *et al.*, 2001;Brown *et al.*, 2013).

Although the importance of root hairs for soil resource acquisition is well known, most research on root hairs has focused on their utility for P and K acquisition, while the value of root hairs for N acquisition has been relatively unexplored. This gap in the literature is a consequence of the fact that the availability of nitrate, the predominant N form in most agricultural soils, is not limited by diffusion (Barber, 1995), and thus N depletion around the root surface is considered to be negligible. Nevertheless, the role of diffusion in limiting overall N acquisition may be significant in soils where ammonium makes up a large fraction of available N. Several studies have reported shifts in root hair length and density in response to suboptimal N availability. For example, Foehse and Jungk (1983) found that tomato, rape and spinach significantly increased root hair length when the nitrate concentration was decreased from 1000 to 2 µm. Robinson and Rorison (1987) reported that root hair density among grass species generally increased as N concentration decreased. While neither N source nor N concentration had significant effects on root hair length in most of these species, *Holcus lanatus* did show an 8-fold increase in root hair length with decreasing nitrate concentration (Robinson and Rorison, 1987). Another study with maize reported a decrease in root hair length under N stress, further suggesting that the response of root hairs to N availability may be species-specific (Gaudin *et al.*, 2011). Using *Arabidopsis* mutants with enhanced and reduced root hair density, Canales *et al.* (2017) demonstrated that plants with denser root hairs accumulated more N from low-N hydroponic solution (which does not accurately mimic N mobility in soil) than plants with sparse or no root hairs. While these observations are suggestive of some relevance of root hairs as part of an adaptive response to N stress, they do not directly explore the utility of root hair length for soil N acquisition.

Similar to their utility for P acquisition, long root hairs could potentially benefit N uptake, particularly in environments where the relative availability of N via diffusion is greater than through mass flow. Modelling has predicted that N uptake by diffusion is especially important under conditions where there is a low rate of transpiration (Phillips *et al.*, 1976). Additionally, a field study in cereals and sugar beet used measurements of water balance in soil strata of varying depth to indirectly estimate that diffusion could contribute up to 85 % of the total N supply to roots, particularly in deep soil strata (Strebel and Duynisveld, 1989). Electrophysiological and molecular evidence demonstrates that root hairs do possess ammonium and nitrate transporters, which are either constitutively expressed or upregulated under low N availability, further pointing to the significance of root hairs as playing a role in N uptake (Lauter *et al.*, 1996;Ludewig *et al.*, 2002).

In this study, we consider the interactions between root hairs and the availability of nitrate and ammonium independently, and we go beyond previous work by testing the potential utility of root hairs for N capture in maize (*Zea mays*) *in silico*, in the field and in realistic media under controlled conditions in a greenhouse. Our goal was to test the hypothesis that long root hairs increase N acquisition by increasing root surface area and expanding the volume of soil explored around the root axis. We address this hypothesis by first utilizing the functional–structural plant model *SimRoot* to determine the relationship between N availability, transpiration rate, and root hair length and density. We follow up *in silico* work with empirical experiments in the greenhouse and field using near-isophenic recombinant inbred lines (RILs) that have similar phenotypes but display variation for root hair length.

## MATERIALS AND METHODS

### *SimRoot* modelling

The functional–structural plant model *SimRoot* is able to integrate parameters of root growth, nutrient uptake and resource allocation from *in vivo* studies to model the relationship between root growth and performance of maize (Lynch *et al.*, 1997;Dathe *et al.*, 2016;Postma *et al.*, 2017). Because *SimRoot* is a heuristic model and not a predictive model, its purpose is to test the adequacy of the proposed hypotheses rather than provide exact predictions of plant growth in the greenhouse or field. To investigate the relationship between root hair phenotypes and N capture under different N and transpiration regimes, *SimRoot* was used to model maize root systems with seven levels of root hair length (0, 0·1, 0·3, 0·5, 0·7, 0·9 and 1 mm), modified by varying the root hair growth rate, and three levels of root hair density (100, 500 and 1000 root hairs cm^−1^). The range of phenotypic variation for root hair length and density parameterized within these models is within the range of natural phenotypic variation observed in maize (Zhu *et al.*, 2005). All other plant properties were held constant in all simulations. For each level of root hair length and density, three levels of nitrate were parameterized: 62·4, 104 and 145·6 kg ha^−1^. Here, N concentration represents the quantity available to the plant in the soil solution, and the buffer capacity of the soil (the ratio between the dissolved and absorbed fraction) is held constant. Transpiration rate was modified at four levels (25, 50, 75 and 100 % transpiration) to manipulate the mass flow component. Starting from germination, plant growth was simulated for 15 d and a resolution of 1 × 1 × 1 mm cubic finite element grid was used. Further information on *SimRoot* is provided by Postma and Lynch (2011). Compiled simulation output for this work is available at https://doi.org/10.5281/zenodo.4073512.

### Greenhouse study

*Plant materials.* To further determine the effect of root hair length on N capture under different sources of N (ammonium sulphate versus potassium nitrate), two greenhouse studies were performed (GHI and GHII). In this work, six RILs from the inter-mated B73 × Mo17 (IBM) population were selected based on their root hair length phenotype previously reported by Zhu *et al.* (2005). RILs descend from the same two parents, hence represent distinct genotypes sharing the same genetic background, thereby reducing the risk of confounding effects from genetic interactions, epistasis and pleiotropy (Zhu *et al*., 2005, 2006). RILs are especially useful tools in cases in which the genetic basis of a phenotype is complex or unknown, as is the case with root hair length and density in maize (Zhu *et al.*, 2005), thereby precluding the use of single-gene variants. These RILs had comparable root and shoot characteristics as evidenced by similar shoot dry weight, root dry weight, total root length and root-to-shoot ratios under non-stress conditions in the greenhouse and similar shoot mass and yield in fertile soil in the field (Supplementary Data Table S1). RILs of the same population have been used to dissect genetic control of root hair traits under differential P availability in maize as well as to investigate the utility of root hair length for P acquisition (Zhu *et al*., 2005, 2010).

*Growth conditions.* The experiments were carried out in a greenhouse located at The Pennsylvania State University, University Park, Pennsylvania (40°48′N, 77°51′W). Two greenhouse trials were performed where the source of N differed, referred to as GHI and GHII. GHI was conducted from March to April 2012 while GHII was conducted from March to April in 2013. In both experiments, seeds were first germinated on germination paper (Anchor Paper Company, St Paul, MN, USA) moistened with 0·5 mm CaSO_4_ in darkness at 28 °C for 3 d. Uniform seedlings were then transplanted into 10·5-L pots (Nursery Supplies, Chambersburg, PA, USA) filled with a mixture of 50 % sand, 35 % vermiculite 5 % perlite and 10 % topsoil (v/v). The topsoil was collected from the Russell E. Larson Agricultural Research Center in Rock Springs, PA, USA (fine, mixed, semiactive, mesic Typic Hapludalf, pH 6·7, silt loam). The soil was incorporated to replicate features found under field conditions, such as the presence of soil biota and organic matter that serves to buffer N availability. The experiment was arranged in a randomized complete block design with four replicates staggered 1 d between replicates. Planting dates and bench locations were considered as block effects. Plants were fertigated with a nutrient solution consisting of (in µm) P (1000), K (3000), Ca (2000), SO_4_ (500), Mg (500), Cl (25), B (12·5), Mn (1), Zn (1), Cu (0·25), Mo (0·25) and DTPA-Fe (25). The pH of the nutrient solution was adjusted to 6.0. Ammonium sulphate ((NH4)_2_SO_4_) was used as the only N source in GHI and potassium nitrate (KNO_3_) was used as the only N source in GHII, and K_2_SO_4_ was used as a supplement for K in the low N treatment. High-N treatments received 7000 µm while low-N treatments received 700 µm of N. Plants were grown under a photoperiod of 14/10 h (light/darkness) at 28/24 °C with total photosynthetically active radiation (PAR) of 1200 µmol photons m^−2^ s^−1^ during the light photoperiod.

### Data collection

Plants were harvested at 35 d after planting (DAP). Shoots were dried at 60 °C for 72 h prior to dry weight determination. The shoots were ground and 2–3 mg of tissue was used for N analysis using an elemental analyser (Series II CHNS/O Analyzer 2400, PerkinElmer). Whole root systems were washed and preserved in 75 % ethanol (v/v) at 4 °C prior to the evaluation of total root length and root hair length. Total root length was obtained by scanning and measuring with image analysis software (WinRhizo Pro, Régent Instruments, Québec City, Québec, Canada). Roots were dried at 60 °C for 72 h prior to dry weight determination.

### Field study

A field study was conducted to further investigate the impact of root hair length under real-world conditions in an agricultural setting. Nine RILs, including the six RILs with varying root hair length characterized in the greenhouse study and three additional RILs, were planted at the Russell E. Larson Experimental Farm of the Pennsylvania State University at Rock Springs, PA, USA (40°42′37″.52 N, 77°57′0″.54 W, 366 masl) from June to October 2012. The soil at the experimental site was a Hagerstown silt loam (fine, mixed, semiactive, mesic Typic Hapludalf). The experiment was a randomized complete block design with a split-split-plot arrangement of treatments and three replicates, with each replicate being an independent 0·4-ha field. RILs were randomly assigned to five-row plots. Each row consisted of 20 plants. The distance between rows was 75 cm and between plants within a row was 23 cm, resulting in a planting density of six plants m^−2^. Based on soil analysis at the beginning of growing season, entire fields were amended with 820 g m^−2^ of sawdust to immobilize soil N. Each field was divided in half to make areas for high and low N. High-N split plots were fertilized with 155 kg N ha^−1^ of urea while low-N split plots did not receive any N fertilizer. Soil nutrient levels of other macro- and micronutrients were adjusted to meet the requirements for maize production as determined by soil tests. Pest control and irrigation were carried out as needed. The plants were harvested at 9 weeks after planting to collect root hair length, leaf and stem biomass and leaf area. Leaf area was estimated from dry weight of leaf discs with 5 cm diameter and total leaf dry weight. Tissue N content was determined using an elemental analyser (Series II CHNS/O Analyzer 2400, PerkinElmer). Soil was collected at 9 weeks after planting for N analysis to a depth of 60 cm. The soil column was divided into four segments: 0–15, 15–30, 30–45 and 45–60 cm depth. The soil was extracted in 2 m KCl and stored at −80 °C. The concentrations of ammonium and nitrate in soil media were determined by spectrophotometry according to Doane and Horwáth, 2003. At maturity grain yield was collected from one full row excluding bordering plants in each plot.

### Root hair evaluation

Three representative root segments were selected from primary and seminal roots (GH) and crown roots (field) of an individual plant. The roots were stained in 0·025 % (w/v) toluidine blue and rinsed in deionized water prior to root hair examination. For determination of root hair length, the stained roots were observed at ×35 magnification under a dissecting microscope (Nikon, SMZ-U, Japan) equipped with a Hamamatsu Photonics XC77 charged-coupled device (CCD) camera. A section of root with consistent fully elongated hairs was selected for image capture. Root hair length was quantified using ImageJ version 1.47 (Abràmoff *et al.*, 2004). Five representative hairs per image were selected for length measurement.

### Statistical analysis

Statistical analyses were performed using R version 2.15.1 (R Development Core Team, 2012). Linear mixed effect models were fitted using the function lme from the package nlme (Pinheiro *et al.*, 2012) and ANOVA was used for comparisons between genotypes and N levels and the interaction between these main effects. A protected least significant difference *post hoc* test (*D* = 0·05) was used for multiple comparisons. Linear regressions were carried out between shoot traits, yield and root hair length in the greenhouse experiments and in the field. Quadratic regression analysis was performed on *SimRoot* output to determine relationships between root hair length and N uptake in different root hair density, N and transpiration levels.

## RESULTS

### Simulation study

**Fig. 1.**
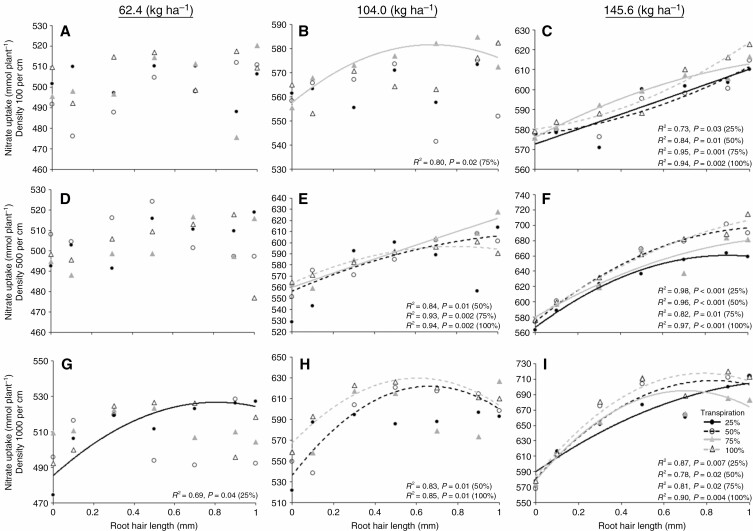
Nitrate uptake by simulated maize root systems when root hair length was varied from 0 to 1 mm. The quadratic-fitted lines represent significant nitrate uptake when the transpiration rate was 25, 50, 75 and 100 % in different N environments. Root hair density was maintained at 100 (A, B, C), 500 (D, E, F) and 1000 per centimetre (G, H, I).

To initially assess the effects of phenotypic variation in root hair length and density on N acquisition, we utilized *SimRoot*, a heuristic functional–structural model focusing on the effects of root phenotypes on soil resource acquisition and plant growth (Lynch *et al.*, 1997;Dathe *et al.*, 2016). The simulation results provided initial support for the hypothesis that root hairs affect N acquisition by suggesting interactions exist between root hair length, root hair density, N availability and N uptake (Fig. 1). In low-N conditions (62·4 kg ha^−1^), greater root hair length significantly enhanced N uptake (*R*^2^ = 0·69) when plants had high root hair density and the transpiration rate was reduced to 25 % (Fig. 1G). The effects of root hair length on N uptake became more pronounced under greater N availability. At intermediate N availability (104 kg ha^−1^), root hair length was correlated with N uptake in phenotypes with low root hair density and 75 % transpiration (Fig. 1B), as it was in phenotypes with intermediate root hair density at all but 25 % transpiration (Fig. 1E) and in phenotypes with high root hair density at 50 and 100 % transpiration (Fig. 1H). Under high N availability (145 kg ha^−1^), root hair length was correlated with N uptake at all transpiration rates (Fig. 1C, F, I).

### Greenhouse study

**Fig. 2.**
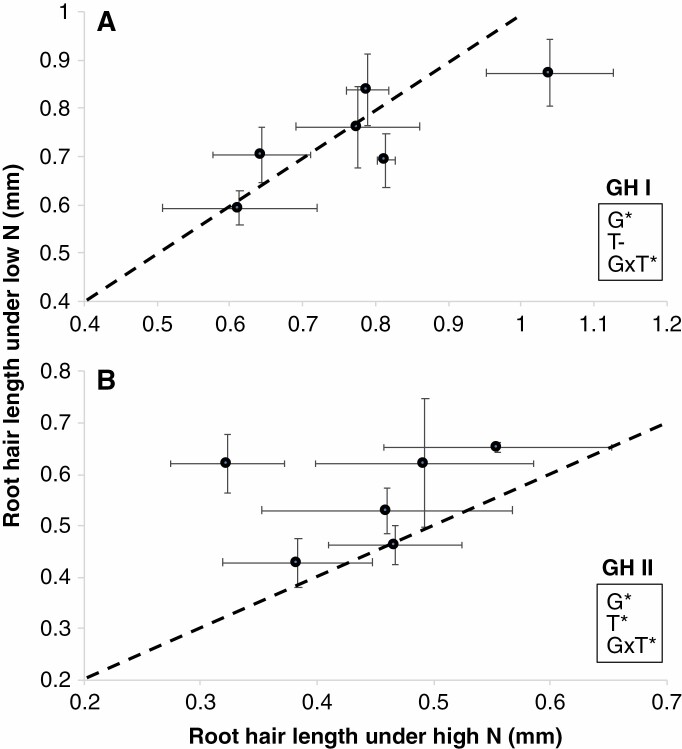
Root hair length of plants grown in the greenhouse experiments under low and high ammonium (GHI) (A) and nitrate conditions (GHII) (B) at 35 DAP. Data shown are means of each RIL with four replicates ± standard errors of the means. The regression line indicates a 1:1 relationship. G signifies genotype, T signifies treatment. *Significant difference at *P* ≤ 0.05.

*Effects of N on root hair length.* In greenhouse experiment I (GHI), ammonium, a relatively less mobile form of N, was provided as the only source of N. Root hair length was significantly different among RILs (*P* ≤ 0.001). However, ammonium stress did not significantly affect average root hair length (Fig. 2A). In greenhouse experiment II (GHII) where nitrate was used as the only source of N, RILs also varied for root hair length and low nitrate availability significantly increased root hair length by an average of 21 % (Fig. 2B).

**Fig. 3.**
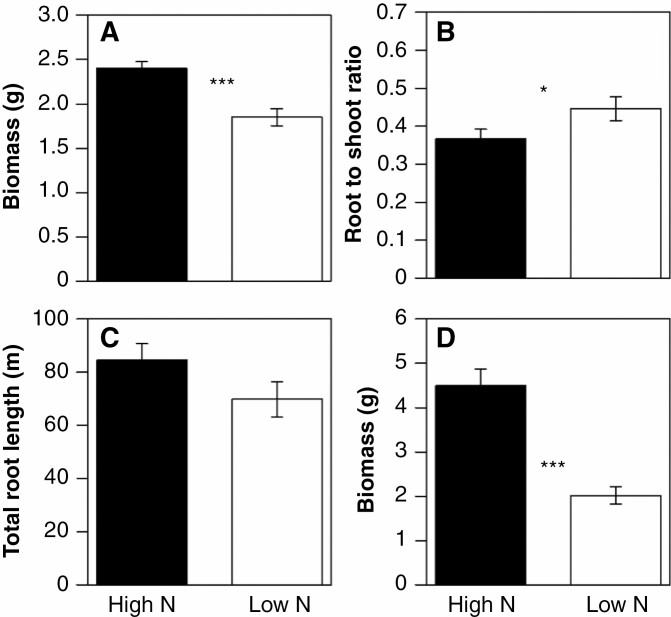
Biomass (A), root-to-shoot ratio (B), and total root length (C) of six maize RILs in GHI and biomass of maize RILs in GHII (D). Plants were harvested at 35 DAP. Data shown are means of four replicates ± standard errors of the means. *Significant difference at *P* ≤ 0.05; ***significant difference at *P* ≤ 0.001.

**Fig. 4.**
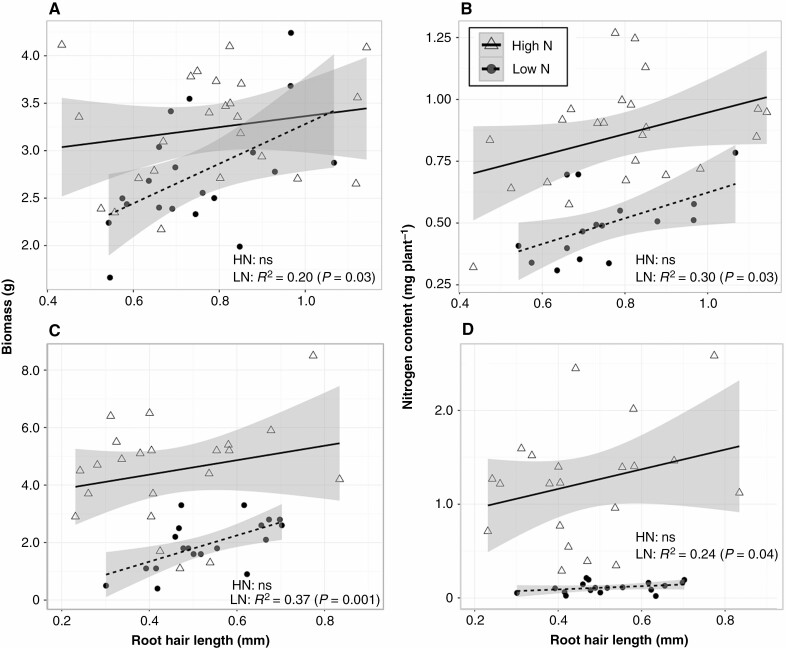
Regression analysis between root hair length and biomass and N content in GHI (A, B) and GHII (C, D) at 35 DAP under high (HN) and low (LN) ammonium (GHI) and nitrate conditions (GHII). Grey shading represents the 95 % confidence interval of the line.

*Effects of N on plant growth.* In GHI, low ammonium availability reduced shoot biomass (dry weight) by 23 % at 35 DAP (Fig. 3A), increased root-to-shoot ratio by an average of 13 % (Fig. 3B), and did not affect total root length relative to conditions of high ammonium availability (Fig. 3C). In GHII, low nitrate availability reduced shoot mass by 55 % relative to the high nitrate treatment (Fig. 3D). When ranges of biomass and plant N content derived from the regression equations were compared, we found that longer root hairs were associated with 44 % greater biomass and 71 % greater plant N content under limited ammonium availability (GHI) (Fig. 4A, B) and 216 % greater biomass and 237 % greater plant N content under limited nitrate availability (GHII) (Fig. 4C, D).

### Field study

**Fig. 5.**
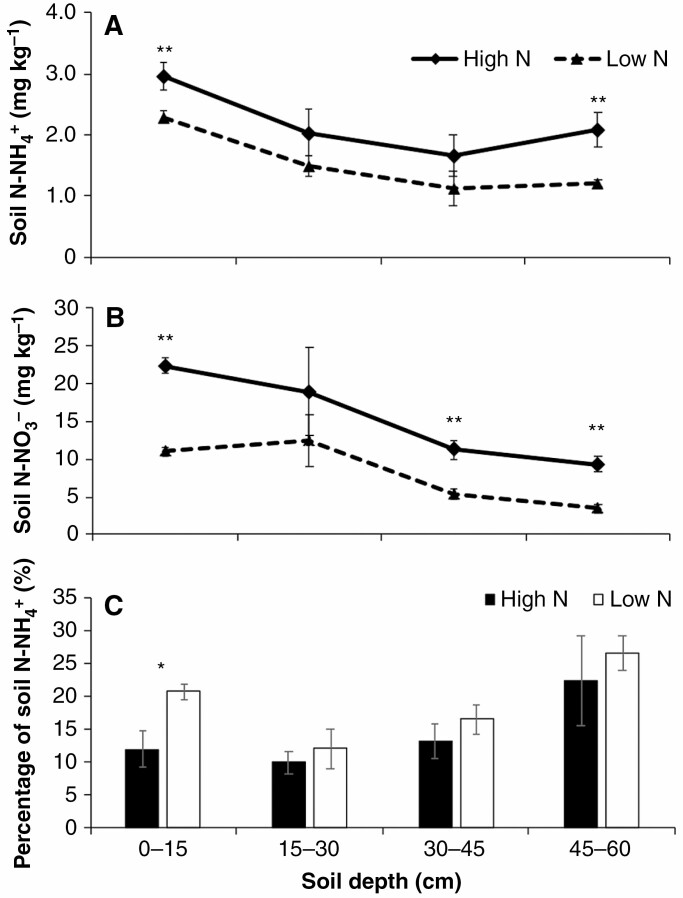
Soil N-NH_4_^+^ (A), N-NO_3_^−^ (B) and the percentage of soil N-NH_ 4_^+^ in total soil available N at different soil depths in the field (C). The soils were collected at 63 DAP from high-N and low-N plots. * and ** represent significant differences between high N and low N at the same soil depth at *P* ≤ 0.05 and *P* ≤ 0.01, respectively.

**Fig. 6.**
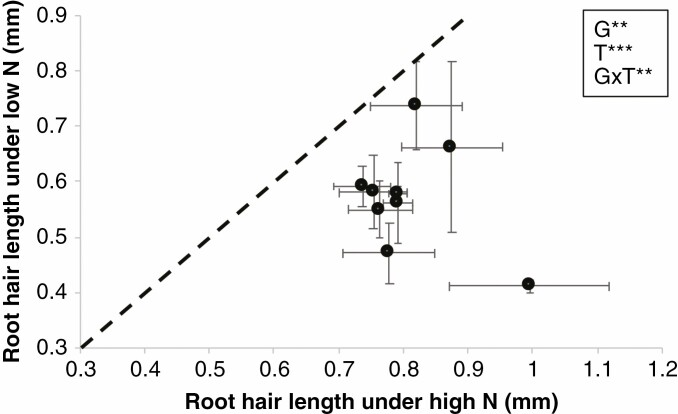
Root hair length of nine maize RILs grown under high-N and low-N conditions in the field. The plants were harvested at 63 DAP. Data shown are means of three replicates ± standard errors of the means. The regression line indicates a 1:1 relationship. G signifies genotype, T signifies treatment. ** and *** represent significant differences between high- and low-N conditions of each RIL at *P* ≤ 0.01 and *P* ≤ 0.001, respectively.

*Soil ammonium and nitrate availability and root hair length.* In the field, ammonium and nitrate were most abundant in the top 15 cm of soil in high-N treatments by 63 DAP (Fig. 5A, B). Nitrate and ammonium availability in the topsoil of high N plots was significantly greater than that of the low N plots (Fig. 5A, B). In the high-N field ammonium represented 11 % of total soil available N in the top 15 cm of soil while the proportion of ammonium in total soil available N was increased to 21 % in low-N soil (Fig. 5C). RILs differed in the phenotypic plasticity of root hair length in response to N availability and the majority of RILs had reduced root hair length by 30 % under low-N conditions in the field (Fig. 6).

**Fig. 7.**
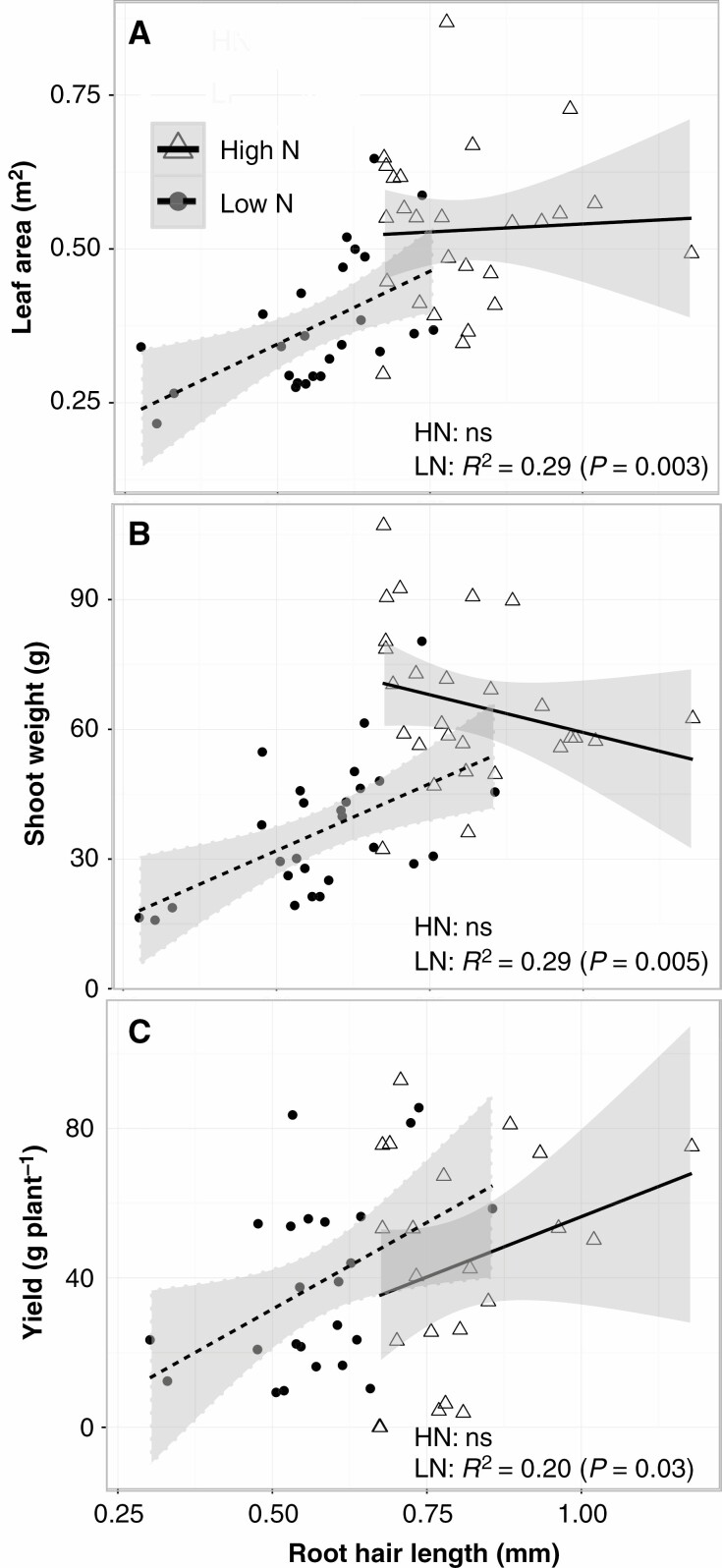
Regression analysis between root hair length and leaf area (A) and shoot weight at 63 DAP (B) and yield (C) under high- (HN) and low-N (LN) conditions in the field. Grey shading represents the 95 % confidence interval of the line.

*Plant growth and yield.* Low N availability decreased shoot biomass at flowering by 45 % and decreased yield by 12 % relative to high-N conditions (Fig. 7). Regression analysis showed that root hair length was significantly associated with leaf area (*R*^2^ = 0.29, *P* ≤ 0.05), shoot biomass (*R*^2^ = 0.29, *P* ≤ 0.05) and yield (*R*^2^ = 0.20, *P* ≤ 0.05) in low-N treatments (Fig. 7). The regression equation between root hair length and plant growth indicated that genotypes with the longest root hairs had 250 % greater shoot mass, 200 % greater plant N content and 267 % greater yield than genotypes with the shortest root hair length under low-N conditions (Fig. 7).

## DISCUSSION

This study investigates the influence of phenotypic variation in root hair length and density in maize on N acquisition by using the functional–structural model *SimRoot* and maize RILs contrasting in root hair length grown under optimal and suboptimal N conditions in the field and controlled conditions. In all three environments, phenotypic variation for root hair length was associated with N acquisition.

### Long root hairs enhanced N uptake under suboptimal N availability

*SimRoot* modelling provided an initial proof of concept in a simplified context and was used to assess the effects of varying transpiration, thereby altering the balance of diffusive flux versus transpiration-driven mass flow. *In silico* results indicate that root hair length and density influence N acquisition, particularly in medium and high N availabilities. Transpiration rate appeared to have little affect at high N availability but became increasingly important at low N availabilities (Fig. 1). Consistent with *SimRoot* results, a positive relationship between root hair length and N acquisition was observed in both greenhouse experiments (Fig. 4), as well as the field trial (Fig. 7). This general agreement among results from *in silico* environments, the field and greenhouse is noteworthy, as each of these environments are distinct. *SimRoot*, like all models, is a simplified representation of the key processes involved in N capture by root systems. The logic structure of *SimRoot* emphasizes spatiotemporal processes regulating soil foraging with a precise accounting of the budgets of C, water, N, P and K acquired from the environment and invested in the construction and maintenance of root tissue, as well as the availability and movement of N in the soil via mineralization, leaching, diffusion and mass flow to roots driven by plant transpiration (*SimRoot* includes a crop canopy module). While *in silico* results suggested an increasing effect of root hair length on N acquisition in environments where N is more abundant (Fig. 1), *in vivo* we observed a significant effect under limited N availability but no effect under high-N conditions (Figs 4, 7). This disparity in results between *in silico* and *in vivo* experiments is not surprising as *SimRoot* is a simplification of reality and, importantly, is never calibrated to force agreement with empirical results as this would violate the premise of heuristic modelling. Our implementation of *SimRoot* in this study did not include several processes that could be important for N capture in the field, including interactions of roots with soil biota, soil physical characteristics apart from water and nutrient mobilities, and rhizosphere modification via root exudates. In a heuristic model such as *SimRoot*, these simplifications are intentional as they permit the testing of the adequacy of the proposed logic model in accounting for empirical observations. In this case, the general agreement of *in silico* and empirical results despite these simplifications indicates that the effects of root hair phenotypes on the spatiotemporal exploration of the soil could account for the positive effects of long root hairs on N capture apart from e.g. microbial effects. Greenhouse mesocosms employ a large physical substrate that has some characteristics of natural soils while permitting greater environmental control, exclusion of root pests and pathogens, and more detailed measurements than are possible in natural soil. Field studies include all of the biophysical and biological complexities of natural soil but are challenging to control and monitor. The fact that each of these three contrasting environments gave similar results reduces the possibility that our conclusions are driven by unusual, artificial and possibly confounding factors unique to any given environment.

Although maize plants grown in the greenhouse did augment their root-to-shoot ratio in response to N limitation, which is consistent with many reports from maize and other plant species (Fig. 3B) (e.g. McConnaughay and Coleman, 1999;Bonifas *et al.*, 2005), there was no significant relationship observed between the proportion of biomass allocation to roots and N uptake under low-N conditions (Supplementary Data Table S2). Additionally, we found no significant relationships between other root traits and plant vegetative growth under N stress (Supplementary Data Table S2), suggesting that the observed relationship between variation in root hair length and shoot mass under limited ammonium and nitrate availability was a consequent effect of root hairs in foraging N. These results are consistent with the hypothesis that longer root hairs enhance N acquisition from the soil, thereby supporting greater vegetative growth and yield than short-haired phenotypes under low-N conditions.

### Root hair length plasticity in response to different environments

Our empirical results indicate that the effects of N stress on root hair length in the greenhouse varied depending on N source (Fig. 2) but that root hair length for most genotypes was significantly decreased by low-N conditions in the field (Fig. 6). The reduction in root hair length in response to N stress in the field is consistent with the study with maize of Gaudin *et al.* (2011) using an aeroponic growth system. The authors hypothesized that reduced root hair length phenotypes observed under low N were indirect responses to decreased plant demand for nutrients as plants had smaller biomass under low-N conditions. However, it is important to note that although there were reductions in root hair length under N stress, the relationships between root hair length and performance were still significant in our study. We speculate that the reduction of root hair length by low N availability is a maladaptive response. Additionally, compared with greenhouse-grown plants, root hair length of field-grown plants was relatively shorter. This result is consistent with other reports in grass species such as rice (Nestler *et al.*, 2016) and maize (Zhu *et al.*, 2010). It is possible that under field conditions the presence of clay and hard particles may limit root hair expansion, and thus root hair length in natural soil is shorter than in artificial sand-based media. Further study investigating the impact of soil types on root hair development will benefit the implementation of root hair traits in crop breeding.

Root hair length of some RILs was not affected by N stress, suggesting that there is variation in root hair length plasticity in response to N. The plasticity of root hair length in response to varying nutrient availability has been shown in many plant species (Foehse and Jungk, 1983;Ewens and Leigh, 1985;Bates and Lynch, 1996;Vatter *et al.*, 2015). Intraspecific genetic variation for root hair length plasticity in response to P stress has similarly been demonstrated in other maize RILs of the IBM population (Zhu *et al.*, 2010). Maize RILs with root hair length plasticity, being short under high P and long under low P, had greater biomass allocation to roots, reduced root respiration and greater shoot biomass under low-P conditions than constitutively long-haired RILs. Analogously, the utility of root hair length plasticity in response to N stress also merits investigation.

### Long root hairs improve N capture possibly by increased root surface area and expanded soil exploration beyond the N depletion zone surrounding the root surface

Nitrate transported to the root surface is thought to be primarily mediated by transpiration-driven mass flow (Barber, 1995). Electrophysiological evidence indicates that high-affinity nitrate transporters in root hairs are greatly up-regulated under nitrate deficiency (Meharg and Blatt, 1995). Increased expression and activity of nitrate transporters could also enhance the rate of nitrate uptake at the root surface, which may exceed the rate of nitrate supply from low-N soil via mass flow of water, creating depletion zones of nitrate. When nutrient uptake occurs due to diffusion and mass flow simultaneously, as in the case of nitrate, investigation of the effect of one of the processes exclusively becomes difficult (Tinker and Nye, 2000). When diffusion limits the motility of a solute in the soil, a depletion zone will develop around the root. When a solute can move through the soil by mass flow, this depletion zone will be reduced or eliminated. However, some studies have suggested that diffusion could be an important mechanism through which nitrate is supplied to the plants (Strebel and Duynisveld, 1989; Jungk, 2001). Modelling of nitrate flux to roots by mass flow and diffusion has been previously described by Phillips *et al.* (1976), who showed that diffusion could be an important mechanism for transport of nitrate to the plant root. However, the model used in that study does not distinguish N uptake from water uptake and there was no independent estimation of diffusion. In the study by Strebel and Duynisveld (1989), in which mass flow and diffusion of N were determined as a function of soil depth and time in many field-grown crop species, the authors concluded that N supplied to the roots by diffusion accounts for an increasing proportion of total N uptake as the growing period proceeds and with increasing soil depth. However, effects of root hairs and means of N supply to roots have never been investigated before. Through our *in silico* study, we are able to address this question by varying mass flow and the transpiration rate to observe the effects of root hair length and density on nitrate uptake under different N availabilities. We observed that with increased root hair density the benefit of longer root hairs was more evident when the contribution of N supply by mass flow was reduced through suppression of transpiration, pointing to the importance of root hairs in soils where N uptake is diffusion-limited (Fig. 1). The fact that we also observed a positive influence of root hair length on N uptake even when transpiration was held at 100 % (100 cm^3^ g^−1^) at medium N availability suggests that the influence of diffusion on N uptake could be larger than previously thought (Fig. 1).

Our empirical results from low ammonium availability in the greenhouse and low N in the field likewise point to the influence of long root hairs in environments where N transport to the root surface is limited by diffusion. In environments in which nitrate is a dominant form of available soil N, overall N uptake may not be limited by diffusion. When the ammonium fraction is high, diffusion may play an important role in overall N uptake. In our field study, the concentrations of ammonium and nitrate in high-N plots at harvest were in the range of typical agricultural soils reported in the literature (1–5 mm nitrate and 0·02–0.2 mm ammonium; Barber, 1995;Owen and Jones, 2001). In the high-N field ammonium represented 11 % of total soil available N in the top 15 cm of soil. Interestingly, the proportion of ammonium in total soil-available N was increased to 21 % in low-N soil. In such conditions, transport of N to roots becomes more diffusion-limited.

### *In silico* results suggest possible competition between root hair length and density

In maize, it has been shown that long root hairs do not incur significant metabolic costs in fertile conditions (Zhu *et al.*, 2010). However, long root hairs and high root hair density could become less important or even detrimental due to greater metabolic and construction costs when nutrient availability is low but not limited by diffusion. For example, Bates and Lynch (2000) demonstrated that under circumstances where P availability is diffusion-limited, the increased gains in P from greater root hair length and density exceeded the metabolic cost of root hairs in *Arabidopsis*. In low-P nutrient solution where P is freely mobile, the benefit of P uptake from root hairs was reduced. These results suggest that there may be a cost for root hair extension under suboptimal availability of nutrients, but those costs are outweighed by the benefits of enhancing P uptake in diffusion-limited environments. Our *SimRoot* results (which do consider metabolic costs) indicate any increase in root hair length increases the root absorptive surface, resulting in greater N uptake at high N availability. However, when N becomes limited high root hair density combined with long root hairs may increase competition with each other and as a result nutrient capture is not proportional to increased root surface area, particularly at high transpiration rates (Fig. 1). This competition probably contributes to the decline in N uptake seen in phenotypes with greater root hair length and density at higher N levels (Fig. 1F, I). Competition among root hairs, however, is challenging to evaluate *in vivo*.

### Strategies for genetic improvement of root hair length and density

Genotypic variation in root hair length and density in maize and common bean is controlled by several important quantitative trait loci (QTLs) (Yan *et al.*, 2004;Zhu *et al.*, 2005;Horn *et al.*, 2016), which suggests that root hair traits could be selected in crop breeding programmes through marker-assisted selection (MAS) as well as through direct phenotyping. Evaluation of root hair phenotypes of a large number of plants can be done effectively and economically in a greenhouse or even in seedlings (Zhu *et al.*, 2005). Moreover, root hair phenotypes can be easily visually evaluated and thus are accessible to crop breeders with limited facilities in developing countries (Vieira *et al.*, 2007). In Mozambique, long and dense root hairs have been incorporated into a common bean breeding programme through direct phenotypic selection, resulting in the release of three new high-yielding P-efficient varieties (Burridge *et al.*, 2019). Combination of long root hairs with other root traits, including shallow growth angle, thin diameter, and root cortical aerenchyma, will allow for efficient topsoil foraging for immobile nutrients including ammonium as well as for mobile resources such as water and nitrate that are not yet taken up by plants or lost to the environment while the primary root is penetrating into deep soil strata early in seedling development. In addition, long root hairs may also benefit early seedling development during the period right after maize fields are fertilized with reduced forms of N fertilizers, such as urea or ammonia (Ribaudo *et al.*, 2011). This is important since long root hairs may help seedlings to establish more quickly than short root hairs, benefiting subsequent growth and development, especially in N-limiting environments. We suggest that long root hairs merit consideration as an avenue to improve N acquisition in maize and other species.

## SUPPLEMENTARY DATA

Supplementary data are available online at https://academic.oup.com/aob and consist of the following. Table S1: root and shoot traits of the RILs under high-N conditions in the greenhouses and in the field. Table S2: correlation coefficients between root traits and vegetative growth and plant N content under low-N conditions in the greenhouse.

mcab104_suppl_Supplementary_Materials
