# Supplementary material for: Root hair phenotypes influence nitrogen acquisition in maize
Source: Ann Bot. 2021 Aug 6;128(7):849–58. doi: 10.1093/aob/mcab104 (PMC8577201; doi:10.1093/aob/mcab104)
Supplement: mcab104_suppl_Supplementary_Materials [file mcab104_suppl_supplementary_materials.docx]

**Supplementary data**

Table S1. Root and shoot traits of the RILs under high N conditions in the greenhouses (n=4) and in the field (n=3). Data shown are mean + SD. Different letters indicate significant difference at p ≤ 0.05 (LSD) while NS indicates that the data are not significantly different.

| **Greenhouse (GHI)** | | | | | |
| --- | --- | --- | --- | --- | --- |
| RILs | Total dry weight (g) | Plant N content (mg plant-1) | Root weight (g) | Root to Shoot ratio | Total root length (m) |
| IBM101 | 2.03+0.34 | 0.56+0.13 | 0.80+0.24 | 0.39+0.06 | 88.25+19.02 |
| IBM106 | 2.24+0.24 | 0.57+0.06 | 0.67+0.12 | 0.29+0.04 | 72.87+4.01 |
| IBM111 | 2.55+0.26 | 0.73+0.11 | 0.72+0.28 | 0.28+0.05 | 80.35+25.58 |
| IBM014 | 2.67+0.14 | 0.68+0.08 | 1.18+0.25 | 0.44+0.11 | 93.98+9.03 |
| IBM199 | 2.35+0.54 | 0.69+0.15 | 1.06+0.17 | 0.47+0.14 | 79.25+6.81 |
| IBM043 | 2.55+0.40 | 0.54+0.22 | 0.75+0.30 | 0.29+0.10 | 89.53+19.83 |
| Significance | NS | NS | NS | NS | NS |
| Greenhouse II (GHII) | | | Field | | |
| RILs | Total dry weight (g) | Plant N content (mg plant^-1^) | Shoot weight (g) | Yield (g plant^-1^) |  |
| IBM101 | 4.76+1.04 | 1.21+0.25 | 58.36+17.53ab | 39.35+13.84 |  |
| IBM106 | 4.5+1.80 | 1.17+0.45 | 96.47+9.35a | 34.71+24.10 |  |
| IBM111 | 4.23+1.08 | 1.28+0.34 | 57.76+0.41b | 50.14+10.05 |  |
| IBM014 | 4.87+0.88 | 1.67+0.45 | 60.20+4.64ab | 68.86+23.65 |  |
| IBM199 | 4.2+0.86 | 2.00+1.05 | 58.78+10.44ab | 44.34+21.26 |  |
| IBM043 | 4.75+0.65 | 1.12+0.28 | 45.83+20.28b | 46.83+9.50 |  |
| IBM007 | - | - | 68.02+10.69ab | 51.68+23.65 |  |
| IBM015 | - | - | 73.60+16.17ab | 38.68+17.68 |  |
| IBM027 | - | - | 73.35+16.90ab | 39.63+13.54 |  |
| Significance | NS | NS | p 0.05 | NS |  |

Table S2. Correlation coefficients between root traits and vegetative growth and plant N content under low N conditions in the greenhouse (GHI). No significant relationships were detected.

| **Root traits** | **Total dry weight (g)** | **Plant N content (mg plant^-1^)** |
| --- | --- | --- |
| **Root dry weight (g)** | 0.11 | 0.02 |
| **Total root length (m)** | 0.05 | 0.002 |
| **Root to Shoot ratio** | 0.15 | 0.05 |
